# Sustained response following BTK inhibitors based treatment in HIV-related primary central nervous system lymphoma: case report

**DOI:** 10.1186/s12981-023-00554-8

**Published:** 2023-08-29

**Authors:** Ying Zhou, Xiaoxue Wang, Xuyong Lin, Jun Wang, Xiaojing Yan, Ying Wen

**Affiliations:** 1 Department of Infectious Diseases II, The First Affiliated Hospital of China Medical University, No. 155, Nanjing North Street, Heping District, Shenyang, 110001 Liaoning Province China; 2 Department of hematology, The First Affiliated Hospital of China Medical University, No. 155, Nanjing North Street, Heping District, Shenyang, 110001 Liaoning Province China; 3 Department of pathology, The First Affiliated Hospital of China Medical University, No. 155, Nanjing North Street, Heping District, Shenyang, Liaoning Province China; 4 Department of neurosurgery, The First Affiliated Hospital of China Medical University, No. 155, Nanjing North Street, Heping District, Shenyang, Liaoning Province China

**Keywords:** Primary central nervous system lymphoma, Human immunodeficiency virus, Bruton tyrosine kinase inhibitor

## Abstract

**Background:**

Despite increasing effort for treating primary central nervous system lymphoma (PCNSL), the prognosis of human immunodeficiency virus (HIV) -related PCNSL was still unsatisfactory. There is currently a lack of clinical evidence for the application of Bruton tyrosine kinase (BTK) inhibitor in HIV-related PCNSL. We reported two HIV-related PCNSL patients, who achieved sustained remission by application of BTK inhibitor based treatment. This protocol had not been previously reported for the treatment of HIV-related PCNSL.

**Case presentation:**

The two cases were characterized by the treatment choice of Bruton tyrosine kinase (BTK) inhibitor. Rituximab was not recommended for them due to their very low CD4^+^ T cell counts. They both took MTX as the first-line therapy and got a relief in initial phase. For the first case, ibrutinib was kept both in the first-line therapy and in the maintenance therapy. When the second case underwent a progressive disease, we continued to use orelabrutinib as one of the salvage treatment, in combination with programmed cell death-1 (PD-1) inhibitor plus lenalidomide. They both achieved a continuous response of up to 20 months without opportunistic infection.

**Conclusions:**

This report highlights the safety and effectiveness of BTK inhibitors, as well as lenalidomide and PD-1 inhibitor in HIV-related PCNSL patients. Both the new therapeutic approaches and a multidisciplinary team authentically contributed to improved survival outcome among HIV-positive PCNSL patients.

## Background

One of the most common HIV-associated lymphomas is diffuse large B-cell lymphoma (DLBCL), including primary central nervous system lymphoma (PCNSL), which generally develops in patients with severe immunosuppression and positive Epstein Barr virus (EBV) [1–3]. The prognosis of HIV-related PCNSL patients is inferior to that in HIV-negative patients [4, 5]. Compared to before the advent of antiretroviral therapy (ART), the overall survival post-ART has improved. Increasing evidence supports induction treatment of high-dose methotrexate (HD-MTX) combination with effective ART in most patients with HIV-related PCNSL [1, 6]. Rituximab has been used successfully in a small number of HIV-related PCNSL patients and other HIV-associated lymphomas [7]. Bruton tyrosine kinase (BTK) inhibitor is promising in PCNSL [8, 9], however its application in HIV-related PCNSL is unclear. Here, we reported two HIV-related PCNSL patients, who achieved sustained remission by application of BTK inhibitor.

## Case presentation

### Case 1

**Fig. 1:**
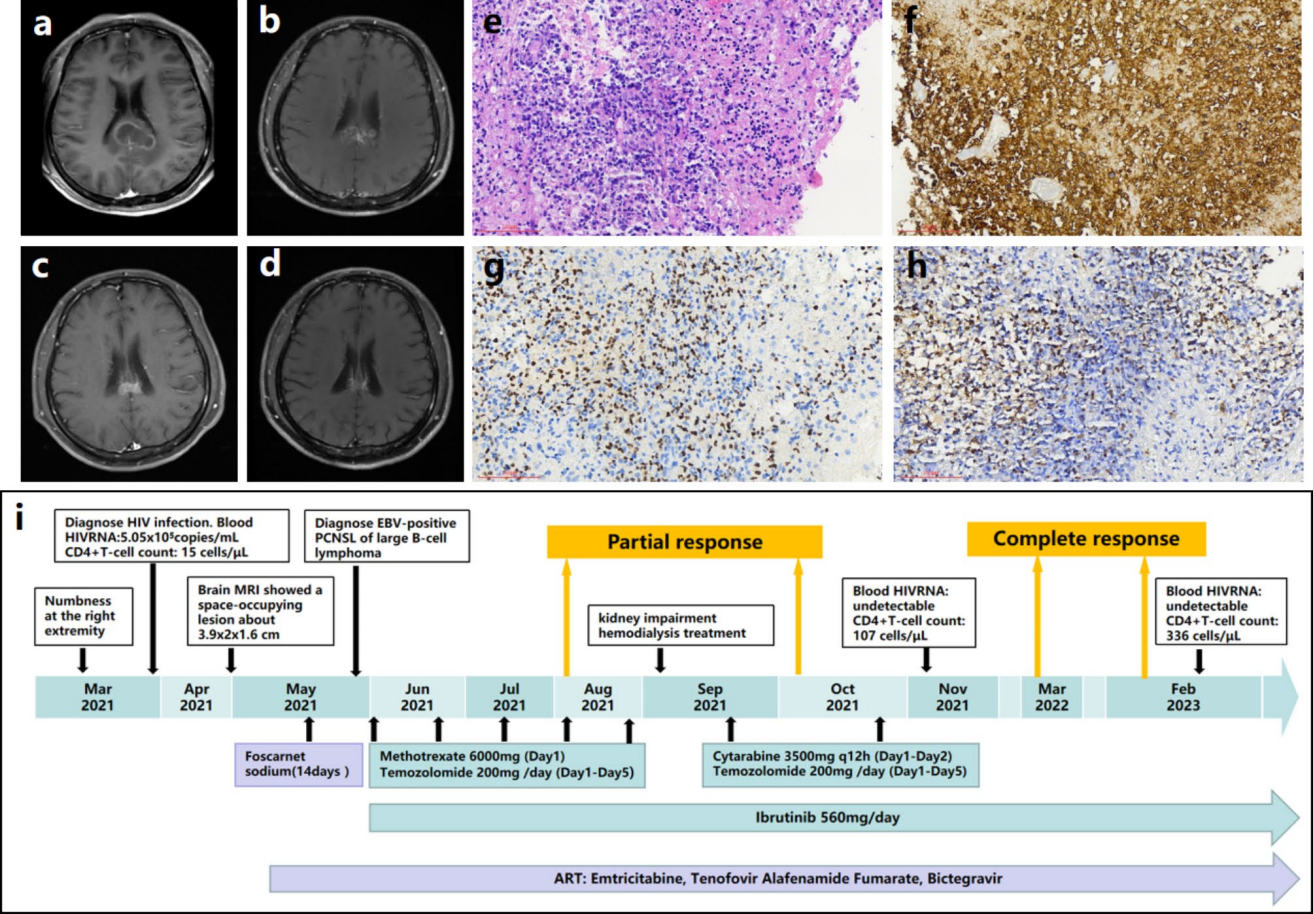
The radiological, pathologic diagnosis and treatment of case 1. Brain contrast-enhanced MRI **(a-d)**: A space-occupying lesion about 3.9 × 2 × 1.6 cm at the corpus callosum with a ring enhancement before treatment (in Apr, 2021) (**a**); Having a partial response (PR) after 2 months treatment (In August, 2021) (**b**); Keeping a PR after 4 months treatment (In October, 2021) (**c**); Achieving a complete response (CR) after 8 months treatment (In March, 2022) and keeping a CR after 19 months treatment (In February, 2023) (**d**). Histological characteristics **(e-h)**: Diffuse distribution of tumor cells with flaky coagulation necrosis were found. There were a large amount of lymphoid cells proliferation especially around the blood vessels and lymphocyte infiltration in blood vessel wall. The volume of some cells is medium to small, and other cells are medium to large. The nucleus is slightly irregular, and nucleolus enlargement and nuclear division were found (H&E staining, original magnification×200) (**e**); The diffuse expression of CD20-positive tumor cells ( immunohistochemical staining, original magnification×200) (**f**). The tumor cells with positive expression of Pax-5 (immunohistochemical staining, original magnification×200)(**g**). The positive EBV-encoded small RNA (EBER) was found (situ hybridization, original magnification×200) (**h**). Treatment regimen adjustment (**i**)

A 41-year-old Chinese man complained numbness at the right extremity for one month in April, 2021. He also had paroxysmal dizziness without nausea, vomiting, convulsions, and consciousness disorder. He had normal muscle strength and muscle tension without sensation dysfunction and neck stiffness. The brain magnetic resonance imaging (MRI) showed a space-occupying lesion (Fig. 1a-d). No extra-cranial lesion was found. HIV confirmatory test was positive. The CD4^+^T cell count was 15 cells/µL. Serum cryptococcal antigen test was negative. T-cell spot of tuberculosis test was negative. Serum EBVCA -IgG and NA -IgG antibodies were positive. EBV DNA was undetectable in peripheral blood and HIV RNA load was 5.05 × 10^5^ copies/mL. The cerebrospinal fluid (CSF) pressure was 150 mm H_2_O, protein level was 936 mg/L, cell count was 12 × 10^6^ /L, acid-fast stain and India ink stain were negative. The cryptococcus antigen, culturing of bacteria and fungus, and cytomegalovirus (CMV) -DNA using CSF samples were also all negative. EBV DNA was 2.662 × 10^4^ copies/mL and HIV RNA loads were 2.06 × 10^5^ copies/mL in CSF samples. No other pathogens were confirmed by the next-generation sequencing in the CSF sample. The biopsy of brain lesions indicated EBV -positive large B-cell lymphoma (not otherwise specified type) (Fig. 1e-h). The foscarnet sodium therapy and ART were initiated. The regimens and treatment course for PCNSL was showed in Fig. 1i. The SMZ/TMP (oral, 2 tablets daily), fluconazole (oral, 200 mg daily) and azithromycin (oral, 200 mg daily) were applied for the prophylactic treatment of Pneumocystis jirovecii pneumonia, fungus infection and mycobacterium infection. After taking the induction regimen of MTX/ibrutinib (a BTK inhibitor) /temozolomide (3 cycles), he experienced a partial response (PR) in August, 2021, then kept the PR though MTX (another 2 cycles) was replaced by Cytarabine (2 cycles) due to its kidney injury adverse effect. He declined the autologous stem cell transplantation. During the maintenance mono-therapy with ibrutinib, he achieved a complete response (CR) in March, 2022. In February, 2023, he still kept CR with undetectable blood HIV RNA and 336 cells/µL of CD4^+^ T cell count.

### Case 2

**Fig. 2:**
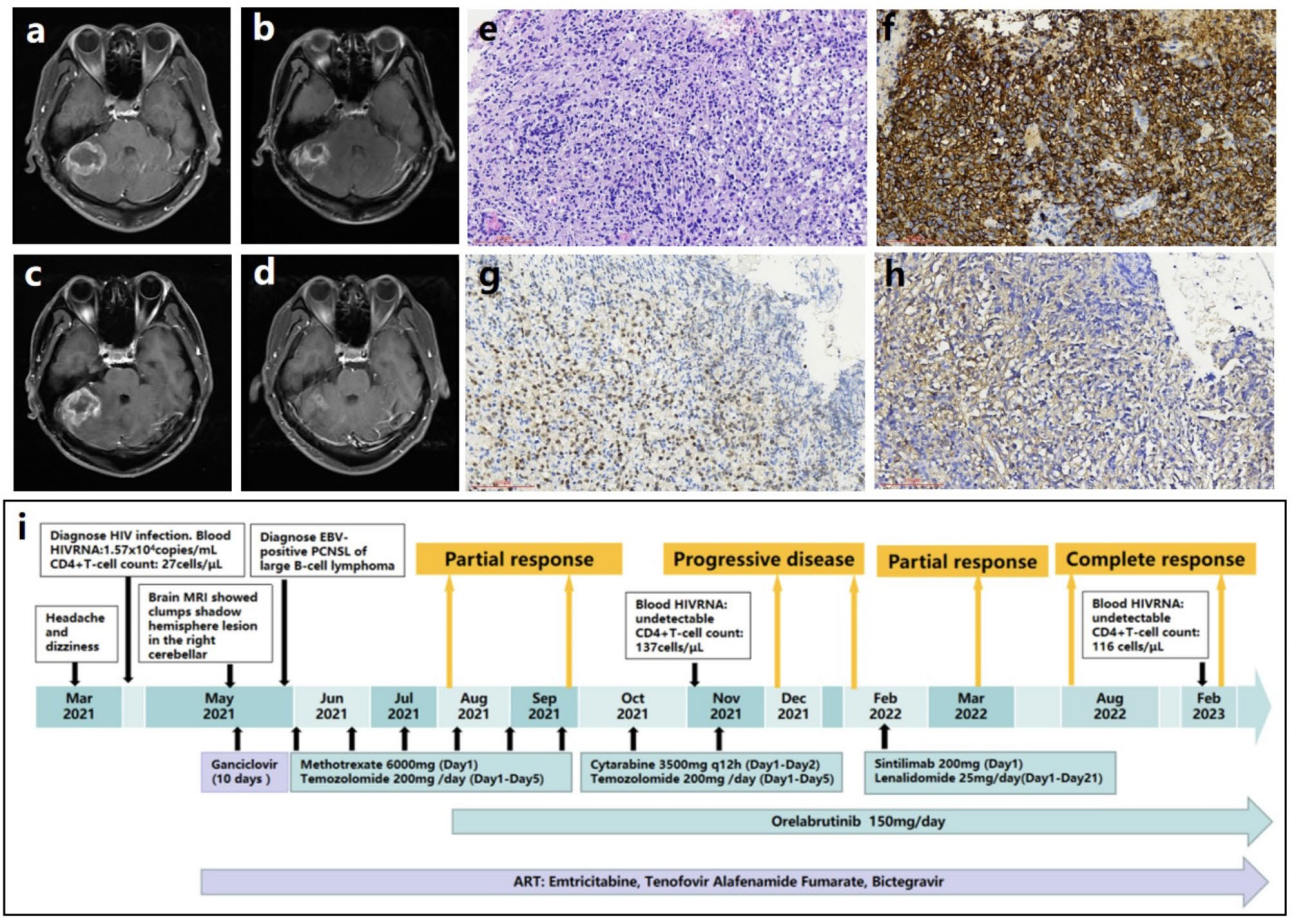
The radiological, pathologic diagnosis and treatment of case 2. Brain contrast-enhanced MRI **(a-d)**: A ring enhancement space-occupying lesion 4.7 × 4.6 × 3.4 cm in the right cerebellar hemisphere with an amygdala cerebelli herniation before treatment (In May, 2021) (**a**). Having a PR after 2 months treatment (In August, 2021) (**b**). Presenting a progressive disease after 6 months treatment (In December, 2021) (**c**). Achieving a CR after 13 months treatment (In August, 2022) and keeping a CR after 19 months treatment (In February, 2023) (**d**). Histological characteristics **(e-h)**: Diffuse distribution of tumor cells with flaky coagulation necrosis was found. The volume of most cells is large. The nucleus is slightly irregular, and nucleolus enlargement and nuclear division were found (H&E staining, original magnification×200) (**e**). The diffuse expression of CD20-positive tumor cells (immunohistochemical staining, original magnification×200) (**f**). The tumor cells with positive expression of Pax-5 (immunohistochemical staining, original magnification×200) (**g**). The positive EBV-encoded small RNA (EBER) was found (situ hybridization, original magnification×200) (**h**). Treatment regimen adjustment (**i**)

A 39-year-old Chinese man complained headache and dizziness for two and a half months in May, 2021. He had no fever, consciousness disorder, convulsions or limbs movement disorder. Mannitol treatment could relieve the symptoms. The brain MRI showed a space-occupying lesion (Fig. 2a-d). No extra-cranial lesion was found. HIV confirmatory test was positive. The CD4^+^T cell count was 27 cells/µL. HIV RNA load was 1.57 × 10^4^ copies/mL in blood. Serum Rubella IgG, CMV IgG, herpes simplex virus-1/2 IgG, EBVCA -IgG, EBNA -IgG antibodies were positive. EBV DNA load was 1.19 × 10^5^ copies/mL in peripheral blood. Serum CMV DNA load was undetectable. T-cell spot of tuberculosis test was negative. To avoid worsening of brain herniation, lumbar puncture was not applied to this case. The biopsy of brain lesion indicated EBV -positive PCNSL of large B-cell lymphoma (non-germinal center B-cell-like type) (Fig. 2e-h). The ganciclovir therapy and ART were initiated. He also adopted the same prophylactic treatment as described in the first case. The regimens and treatment course for PCNSL was showed in Fig. 2i. After taking the induction regimen of MTX/temozolomide (3 cycles), he got a PR in August, 2021, and kept PR with MTX/temozolomide/orelabrutinib (a BTK inhibitor) (another 3 cycles). He declined the autologous stem cell transplantation. Then he had a disease progression (PD) during the regimen of Cytarabine/temozolomide/orelabrutinib (2 cycles) in December, 2021, and adopted the salvage treatment regimen of sintilimab (PD-1 inhibitor) /orelabrutinib/lenalidomide (an immunomodulatory drug) followed by another PR in March, 2022. In August, 2022, he experienced a CR during orelabrutinib/lenalidomide maintenance therapy. Then, the patient continued mono-therapy of orelabrutinib and still kept CR with undetectable blood HIV RNA and 116 cells/µL of the CD4^+^T cell count until the lasted follow-up in May, 2023.

## Discussion

The influence of ART on HIV-related PCNSL was beneficial and ART could really prevent from opportunistic infections occurrence after virological control and immunological recovery. The efficacy of anti -EBV therapy on PCNSL was undetermined [10]. There is currently no standard treatment for HIV-related PCNSL, and national comprehensive cancer network guidelines recommend the application of HD -MTX as induction therapy [11]. These two cases took MTX as the first-line therapy and got a relief in initial phase. Rituximab was not recommended for these two cases due to their very low CD4^+^T cell counts. However, the first case stopped MTX during induction therapy due to its kidney injury adverse effect and the second case got a progressive disease during consolidation therapy. For relapsed or refractory PCNSL, BTK inhibitors and immunomodulatory drugs such as lenalidomide and pomalidomide can be effective [8, 9]. PD-1 inhibitors suggest better efficacy in a small sample of retrospective studies for relapsed/refractory HIV-negative PCNSL [12, 13]. However, the effectiveness of these treatments in HIV-related PCNSL is uncertain. In our first case, ibrutinib has added to first-line therapy and achieved further remission by maintenance therapy, which showed similar response for treatment -naïve HIV-negative PCNSL cases [14, 15]. The ibrutinib/MTX combination followed by ibrutinib mono-therapy maintenance achieved 64% of CR [14]. The ibrutinib/MTX /temozolomide combination achieved 88.9% of CR [15]. In the second case, orelabrutinib, PD-1 inhibitor plus lenalidomide acted as the salvage treatment and achieved another PR after disease progression and then orelabrutinib achieved sustained remission as maintenance treatment, which showed similar response for relapsed/refractory HIV-negative PCNSL cases [16, 17]. The orelabrutinib/lenalidomide -containing combination therapy achieved 73.3% of CR [16]. Although PD-1/ programmed death ligand 1 (PD-L1) over-expression in the micro-environment of PCNSL suggested a therapeutic recommendation, the therapeutic response and prognostic correlation was inconclusive [18–20]. The relapsed/refractory PCNSL cases with/without gene alterations (such as MYD88) of B-cell receptor signaling pathway demonstrated the clinical response to BTK inhibitors [21]. From PCNSL diagnosis until now, the overall survival of both patients were 20 months, which suggested the efficacy of BTK inhibitors, as well as lenalidomide and PD-1 inhibitors. Both patients did not have drug side effects and opportunistic infections occurrence throughout the treatment, which suggested the safety of BTK inhibitors and other target therapy. The efficacy of the BTK inhibitors monotherapy for PCNSL was limited [22, 23]. In order to avoid BTK inhibitors resistance and augment the BTK inhibitors response, it was suggested that BTK inhibitors was added into combination regimen. Last but not least, the multidisciplinary team including HIV specialist, neurosurgeon, pathologist and hematologist demonstrated the diagnostic and therapeutic advantages for HIV-positive PCNSL patients.

## Data Availability

All data generated or analyzed during this study are included in this published article.

## Abbreviations

DLBCL: Diffuse large B-cell lymphoma
PCNSL: Primary central nervous system lymphoma
BTK: Bruton tyrosine kinase
PD-1: Programmed cell death-1
PD-L1: Programmed death ligand 1
HIV: Human immunodeficiency virus
MRI: Magnetic resonance imaging
ART: Antiretroviral therapy
HD -MTX: High-dose methotrexate
CSF: Cerebrospinal fluid
EBV: Epstein Barr virus
CMV: Cytomegalovirus
PR: Partial response
CR: Complete response
